# Cimicifuga, in Its Relation to Acute Catarrh

**Published:** 1882-05

**Authors:** C. Carleton Smith

**Affiliations:** Philadelphia


### CIMICIFUGA IN ITS RELATION TO ACUTE CATARRHAL SYMPTOMS.

C. Carleton Smith, M. D., Philadelphia.

1. Sneezing: which in half an hour becomes quite frequent.

Obstruction of *left* nostril in the evening.

Pain in head for *ten days*, followed by coryza with sore throat, and gradual extension to the bronchial mucous membrane; and this followed by short, dry, hacking cough, night and day, for *two weeks*.

Constant coryza all day, causing *smarting* and *burning*, and *aching* in the nose.

*Watery* coryza, which becomes very abundant, which finally becomes *greenish*, and slightly sanguineous.

Other provers observed first, a *dry*, *stuffed* condition of the nostrils, followed by profuse watery discharge, with extreme sensitiveness to cool air, as if the base of the brain were laid *bare*, and every inhalation brought the cold air in contact with it.

### Face.

1. *Flushed* and *red* face with pain in under-jaw, lower teeth, and *right superior* maxillary bone.

2. Lips *dry* and *feverish*, even *sore*.

### Eyes.

1. *Inflammation* of the *eyelids*, with *pain* and *great soreness;* every motion of the eyeballs painful, *especially on looking upward.*

2. The eyeballs *ache* severely, especially the *right* one. Pain runs from eyes up to *top of head;* the pain in head produces soreness throughout the brain, aggravated by *every motion.*

### Throat.

1. Great *dryness of throat*, with a constant sensation of *fullness* in *pharynx.* (This *fullness* is a *constant symptom.*)

2. Increase of *thick viscid mucus* in the *fauces.* (*Merc. Iod.*) with inclination to swallow. (*Merc. Iod.* has inclination to hawk this mucus up.)

### Respiratory Organs.

1. Constant inclination to cough, from tickling in larynx, aggravated by speaking. This constant inclination to cough, almost *prevents speaking.*

2. Hoarseness with *roughness* and *scraping* of the throat: dry, hacking cough night and day, for *two weeks* in *succession.*

3. *Breath* very *offensive.*

4. *Difficult respiration* after walking, or the *least exertion.*

### Neck and Back.

1. Stiffness of neck, with *rheumatic* pains in the muscles of the neck; a feeling of contraction.

2. Terrible pain and aching in *lumbar region,* which is better from pressure.

Tired feeling in back, extending from *region of kidneys* to *sacrum.*

### Extremities.

1. An uneasy feeling amounting to an *ache*, through *all* the *extremities,* every time the dose was taken. A feeling in limbs and joints, as if he had *labored hard all* day.

### General Symptoms.

1. Restlessness very great, desires to *move about continually;* does not know *what to do,* or *where* to *go. Cannot fix his mind on anything.*

This latter is a symptom similiar to *Ars.*, and taking this as a key-note, we might be led to give that drug, and thereby fail to cure our patient. Under *Cim.* the patient is very restless *at night*, but *not particularly* after *midnight*, as under *Ars.*

*General* feeling of *exhaustion* and a disposition to diarrhœa after getting up in the morning.

REMARKS.

Sleeplessness of *Cimicifuga* closely resembles *Aconitum*, but *Acon.* sleeplessness, like *Ars.*, is worse after *midnight*, while the *Cim.* sleeplessness runs through the whole night. The patient also complains of *numbness* under *Cimicifuga*, which prevents his sleeping.

*Cimicifuga* resembles both *Bry.* and *Puls.* in rheumatism.

Also resembles *Caulophyl.* in rheumatic uterine affection.

